# Lymphatic filariasis control in Tanga Region, Tanzania: status after eight rounds of mass drug administration

**DOI:** 10.1186/s13071-014-0507-5

**Published:** 2014-11-12

**Authors:** Paul E Simonsen, Yahya A Derua, Stephen M Magesa, Erling M Pedersen, Anna-Sofie Stensgaard, Mwelecele N Malecela, William N Kisinza

**Affiliations:** Department of Veterinary Disease Biology, Faculty of Health and Medical Sciences, University of Copenhagen, Dyrlægevej 100, 1870 Frederiksberg C, Denmark; National Institute for Medical Research, Amani Medical Research Centre, P.O. Box 81, Muheza, Tanzania; RTI International, Global Health Division, Dar es Salaam, Tanzania; Center for Macroecology, Evolution and Climate, Natural History Museum of Denmark, University of Copenhagen, Universitetsparken 15, 2100 Copenhagen Ø, Denmark; National Institute for Medical Research, P.O. Box 9653, Dar es Salaam, Tanzania

**Keywords:** Lymphatic filariasis, *Wuchereria bancrofti*, Control, Mass drug administration, Ivermectin, Albendazole, Microfilariae, Circulating filarial antigens, Vectors, Tanzania

## Abstract

**Background:**

Lymphatic filariasis (LF) control started in Tanga Region of Tanzania in 2004, with annual ivermectin/albendazole mass drug administration (MDA). Since then, the current project has monitored the effect in communities and schools in rural areas of Tanga District. In 2013, after 8 rounds of MDA, spot check surveys were added in the other 7 districts of Tanga Region, to assess the regional LF status.

**Methods:**

LF vector and transmission surveillance, and human cross sectional surveys in communities and schools, continued in Tanga District as previously reported. In each of the other 7 districts, 2–3 spot check sites were selected and about 200 schoolchildren were examined for circulating filarial antigens (CFA). At 1–2 of the sites in each district, additional about 200 community volunteers were examined for CFA and chronic LF disease, and the CFA positives were re-examined for microfilariae (mf).

**Results:**

The downward trend in LF transmission and human infection previously reported for Tanga District continued, with prevalences after MDA 8 reaching 15.5% and 3.5% for CFA and mf in communities (decrease by 75.5% and 89.6% from baseline) and 2.3% for CFA in schoolchildren (decrease by 90.9% from baseline). Surprisingly, the prevalence of chronic LF morbidity after MDA 8 was less than half of baseline records. No infective vector mosquitoes were detected after MDA 7. Spot checks in the other districts after MDA 8 showed relatively high LF burdens in the coastal districts. LF burdens gradually decreased when moving to districts further inland and with higher altitudes.

**Conclusion:**

LF was still widespread in many parts of Tanga Region after MDA 8, in particular in the coastal areas. This calls for intensified control, which should include increased MDA treatment coverage, strengthening of bed net usage, and more male focus in LF health information dissemination. The low LF burdens observed in some inland districts suggest that MDA in these could be stepped down to provide more resources for upscale of control in the coastal areas. Monitoring should continue to guide the programme to ensure that the current major achievements will ultimately lead to successful LF elimination.

## Background

Human lymphatic filariasis (LF) is a disfiguring and disabling mosquito-borne parasitic disease [1]. It is a major health problem in many warm climate countries and one of the most prevalent of the so-called Neglected Tropical Diseases (NTDs). The causative filarial parasites are transmitted to humans when female mosquito vectors carrying infective larvae land on the human skin to take a blood meal. The larvae penetrate the skin and migrate to the lymphatic vessels where they develop into adult male and female worms over a period of several months. The mature fertilized female worms produce large numbers of tiny larvae called microfilariae (mf) which circulate in the blood. The mf are ingested by new mosquito vectors when taking a blood meal and develop into new infective larvae in the vectors in about 10–14 days. Clinical LF disease (e.g. acute filarial fever, lymphedema, elephantiasis, hydrocele) primarily results from damage caused by the adult worms in the lymphatic vessels. The disease manifestations can be a cause of considerable incapacity to the affected individuals, and LF has been recognized as one of the leading causes of long-term disability in the world [2].

The World Health Organization launched a Global Programme to Eliminate Lymphatic Filariasis (GPELF) in 2000 with the major goal to eliminate LF as a public health problem [3,4]. GPELF provides guidance and support to national LF control programmes, and the principal intervention measure currently recommended is annual mass drug administration (MDA) with a two-drug combination to LF endemic communities. In most countries a combination of diethylcarbamazine (DEC) and albendazole is used, but due to the risk of serious adverse reactions in individuals infected with *Onchocerca volvulus*, the combination of ivermectin and albendazole is used in African countries which are co-endemic for these infections [3,4]. The drugs are mainly microfilaricidal and the main purpose of the MDA is to kill circulating mf and thereby to reduce transmission. By preventing new infections, this strategy should eventually lead to elimination of LF infection in the population [4,5].

In Africa, where all LF is caused by the filarial nematode *Wuchereria bancrofti*, estimates indicate that more than 45 million people are affected [6]. Tanzania ranks as the third African country in terms of highest LF burden, with over 34 million people living in endemic areas, and more than 6 million infected [7]. Particularly high prevalences have been reported from the coastal zone along the Indian Ocean [8-13]. As one of the first countries in Africa, Tanzania launched a National Lymphatic Filariasis Elimination Programme (NLFEP) in 2000, and the first MDA was implemented in endemic areas of Coast Region near Dar es Salaam in the same year. Since then, the NLFEP has year by year expanded its geographical coverage, by applying annual MDA with a combination of ivermectin (150–200 μg/kg body weight) and albendazole (400 mg/person) to individuals aged ≥5 years in LF endemic districts. In principle, the community-directed intervention approach [14] is used for delivering the drugs, although sometimes a more top-down delivery mechanism is applied [15]. Since 2009, the NLFEP has been integrated with the Neglected Tropical Diseases Control Programme, whereby the MDA for LF control is coordinated with MDA programmes for other NTDs prevailing in the same districts.

During 2004–2011 the present study monitored the effect of the NLFEP MDA activities on LF transmission and human infection in communities and primary schools in rural areas of Tanga District [16-18]. It was seen that the level of transmission and human infection initially decreased considerably, but also that the effect leveled off and became less pronounced in subsequent years. Transmission was still ongoing after 6 MDAs although at a much reduced level. For logistical reasons, most monitoring activities were suspended in 2012, with exception of two months of vector and transmission surveillance. However, in 2013, the surveys for infection and disease were resumed in the same communities and schools as previously. In addition it was decided to add spot check surveys in the other 7 districts of Tanga Region covered by the LF control programme, to assess the overall regional LF status after 8 rounds of MDA.

## Methods

### Study area

All study sites were located in rural areas of Tanga Region (approx. 2,045,000 inhabitants in 2012) in north-eastern Tanzania. The region has 8 districts - Handeni, Kilindi, Korogwe, Lushoto, Mkinga, Muheza, Pangani and Tanga – of which the latter includes the regional capital of Tanga City (approx. 270,000 inhabitants). From the coastal zone along the Indian Ocean the land gradually rises to a plateau in the hinterland with an altitude of about 500 meters. From this plateau, the Nguu mountains (in the western part of Kilindi District), and the Usambara mountains (in parts of Muheza, Lushoto and Korogwe districts) further rise to altitudes of about 1500 meters and >2000 meters, respectively. The region has two rainy seasons in the year, the long rains from March to June and the less intensive short rains from November to December. A map indicating the location of the study sites in Tanga District is given in [18].

### LF control activities in Tanga Region

**Figure 1:**
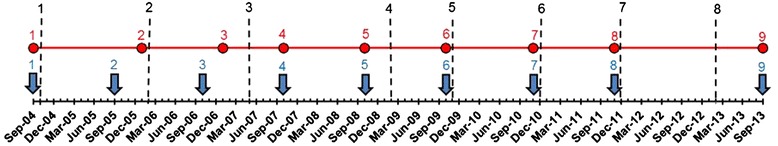
**Timing of MDA and survey activities in rural study communities and schools in Tanga District between 2004 and 2013.** Black vertical stippled lines = MDA; Red circles = community surveys; Blue arrows = school surveys. Figures above symbols = activity number.

In 2004, all districts of Tanga Region were enrolled in the NLFEP and received the first MDA with ivermectin and albendazole. Following integration of the NLFEP with the Neglected Tropical Diseases Control Programme in 2009, the MDAs for LF control basically continued as before, but activities were coordinated with school based praziquantel treatment for schistosomiasis and community based treatment with azithromycin for trachoma (the latter two treatments given two weeks after the MDA for LF control). The 8 MDAs for LF control administered during the period covered in the present study were given in October 2004, February 2006, May 2007, February 2009, November 2009, December 2010, December 2011 and February 2013 (Figure 1), and thus had time intervals of 16, 15, 21, 9, 13, 12 and 14 months. Before start of LF control in Tanga Region, three MDAs with ivermectin for control of onchocerciasis had been implemented in highland areas of Muheza, Lushoto and Korogwe districts (in 2001, 2002 and 2003).

### Design of community study in Tanga District

Vector and transmission surveillance started in all four administrative sections (hamlets) of Kirare village (located about 20 km to the south of Tanga City) in beginning of November 2003, i.e. about one year before the first MDA, and continued uninterrupted until the end of December 2011. The surveillance was resumed for a two-month period in 2012 (June/July) and 2013 (May/June) towards the end of the long rainy season (when highest vector density had been noted during previous years) by using same catching houses and methods.

A pre-MDA cross-sectional survey of the human population in all four hamlets of Kirare was carried out in September 2004 (survey 1), immediately prior to the first MDA (Figure 1). All individuals aged ≥1 year were examined for microfilariae (mf) and chronic clinical manifestations of LF, and volunteers from mosquito collection houses had venous blood samples collected and examined for circulating filarial antigen (CFA) by ELISA. Subsequent similar surveys were carried out in Kirare in January 2006, January 2007, October 2007, October 2008 and October 2009 (surveys 2–6; Figure 1). In the following three surveys (7 in November 2010, 8 in November 2011 and 9 in September 2013; Figure 1) some changes were made. First, only two of the four hamlets comprising Kirare village (Mtambuuni and Mashine) were included. As substitutes, and in order to cover a larger geographical area, one hamlet from each of two other villages in Tanga District (Mabavu in Kiomoni village about 5 km north-west of Tanga city, and Majengo in Kisimatui village about 17 km south-west of Tanga city) were included. Second, all individuals were first screened for CFA with rapid ICT card tests during the day, and only those positive for CFA were screened for mf at night (no venous blood samples taken). The examination methods described in the following refer to those used in survey 7–9, whereas methods used in earlier surveys have been described previously [16,18].

### Design of school study in Tanga District

The new Standard 1 pupils (most being 6–7 years of age) entering 10 rural primary schools each year were examined for CFA (Figure 1), as previously described [17,18]. Briefly, with assistance of the teachers, and by following the school registers, the pupils were screened for CFA by use of rapid ICT card tests. The schools were in four clusters located south-west (Kirare, Mapojoni and Marungu; no. 1–3), north-west (Kiomoni and Mafuriko; no. 4–5), close west (Maweni and Kange; no. 6–7) and more distant west (Pongwe, Kigandini and Ziwani; no. 8–10) of Tanga city. The distance of the schools to the centre of Tanga city ranged from 5 to 24 km.

### Design of spot check surveys in other districts of Tanga Region

In each of the other 7 districts of Tanga Region, 2–3 villages were purposively selected for spot checks which were carried out in October/November 2013. Sites were selected to spatially adequately cover the region and in areas where the environment appeared to be supportive to transmission of LF. At each site, approx. 200 pupils from the local primary school were screened for CFA with rapid test cards, by following the school registers and with the assistance of the teachers. Screening started with pupils in Standard 1, and if these were not enough it continued with those in Standard 2, 3, etc., until approx. 200 were reached. At 1–2 of the sites in each district approx. 200 community members aged ≥10 years were requested to come for CFA screening with rapid test cards, and those ≥15 years were clinically examined for chronic signs of LF. CFA positive community members moreover had a finger-prick blood sample examined for mf in the evening.

### Ethical considerations

Meetings were held regularly with the study communities in Tanga District, and prior to community spot checks in other districts, to inform the inhabitants about study contents and to obtain their cooperation. Before any examination, individuals were asked if the purpose and consequences as explained during the meetings had been understood, questions for clarifications were answered, and written consent to participate was obtained (from adults, and from parents/guardians of individuals less than 18 years old).

Prior to each years’ school surveys in Tanga District, meetings were held with the head teacher, the relevant teachers and the parents committee for the individual schools, to inform about the contents of the study. The members of the parents committee provided written informed consent, on behalf of the children and their parents. A similar procedure was followed during the school spot checks in the other districts. Children who refused to participate in the examinations were not included.

Individuals were informed about their mf or CFA status after the surveys, and those found positive were particularly encouraged to participate and take the drugs during the next MDA. Ethical and research clearance to the study was provided by the Medical Research Coordinating Committee of the National Institute for Medical Research in Tanzania.

### Examinations for LF infection and disease

Schoolchildren and community members were examined for CFA status by use of rapid immunochromatographic test cards (ICT cards, Binax NOW Filariasis, Inverness Medical Innovations Inc., USA), by following the manufacturers’ instructions. Briefly, one hundred microliters of finger prick blood were applied to the sample pad on the test card, and the result was read after 10 minutes as either positive or negative.

Blood was examined for mf by using the counting chamber technique [19]. From each individual, 100 μl of finger prick blood was collected into a heparinized capillary tube after 21:00 hours (due to the nocturnal mf periodicity in the study area) and transferred into a microtube with 900 μl of 3% acetic acid. Later in the laboratory the specimens were transferred to a counting chamber and examined for mf under a compound microscope. The specimens were examined blinded by two different technicians, and the mean count was used as the mf intensity.

During community surveys all study individuals were examined for chronic manifestations of LF by a clinical officer. Manifestations were graded as described previously [10], but in this presentation lymphoedema/elephantiasis ≥ grade I (loss of contour, pitting oedema) and hydrocele ≥ stage II (true hydrocele ≥6 cm, with fluid accumulation) are reported as elephantiasis and hydrocele, respectively.

### Vector and transmission surveillance

Mosquitoes were collected from 50 originally randomly selected households in Kirare using Centre for Disease Control light traps (John W. Hock Company, Gainesville, USA) hung beside an occupied bed with an untreated bed net. Mosquitoes were collected from each of the selected households once weekly (10 houses sampled on the night of each weekday), as described [16,18]. Traps were switched on at 18:00 hours and off at 06:00 hours by trained field assistants. Caught mosquitoes were transferred to paper cups and transported to the laboratory in Tanga for identification using morphological criteria. The live female vectors (*An. gambiae*, *An. funestus* and *Cx. quinquefasciatus*) were dissected under microscope for larvae of *W. bancrofti*, as described before [13].

### Questionnaire surveys for treatment coverage and bed net use

Questionnaire surveys were carried out in the study communities and study schools in Tanga District shortly after each MDA to assess the treatment coverage. In these surveys, individuals were asked in privacy whether or not they had taken the drugs (in communities, parents answered on behalf of their children below 15 years of age). In November 2013, a more elaborate questionnaire survey was carried out among individuals identified to be mf positive during community survey 9, to assess their views and adherence with the MDA programme.

Very few households possessed bed nets at the start of the study in 2003, but bed nets were observed to gradually become more common during the study. In September 2011, insecticide treated nets (ITNs) were moreover distributed to every household in Tanga Region [18]. During the latter community post-MDA questionnaire surveys, heads of households were therefore also asked about the number and type of bed nets possessed in their household (checked by visual inspection when inhabitants allowed). During the post MDA 7 survey in January 2012, 97.6% of 380 households indicated they possessed at least one bed net (96.6% an ITN), and during the post MDA 8 survey in February 2013, 81.1% of 392 households possessed at least one net (70.7% an ITN).

### Mapping

Longitude and latitude geo-coordinates of study sites were recorded with a hand held GPS (eTrex, Garmin). Maps showing prevalence at individual study sites in Tanga Region were prepared in the open source geographical information system Qgis (version 2.0). To obtain an indication of prevalence zones beyond the individual sampling locations, kriging, which quantifies unknown values from known values based on their respective distance and direction, was considered for the school spot check CFA data. Data trends and distribution was first explored using ArcGIS 10.1 Geostatistical Analyst (ESRI, Redlands, CA) to ensure the most accurate geostatistical interpolation method was applied. Empirical Bayesian kriging was chosen as the favored interpolation method, as it is considered more accurate than ordinary kriging for small datasets and due to its ability to account for the error introduced by estimating the underlying variogram [20]. Parameters were slightly adjusted to give the best model fit to the empirical data (standardized mean nearest to zero, smallest root-mean-square prediction error, average standard error nearest the rootmean-square prediction error, and standardized root-mean-square prediction error nearest to one).

### Data analysis

Although community surveys in Tanga District also included younger individuals, only those aged ≥10 years and ≥15 years were included in analysis of LF infection and LF chronic disease, respectively, in order to allow easy comparison of findings to those from community surveys in the other districts (only little infection and chronic disease is seen in individuals below these age groups).

The mf intensities were adjusted for sampling time by multiplying the counts with a time-specific factor, as previously described [21]. Geometric mean intensities (GMIs) of mf counts were calculated as antilog [(Σ log x +1)/n] – 1, with x being the mf/ml and n the number of individuals included. During Tanga District community surveys 7–9, and during the community spot check surveys in the other districts of Tanga Region in 2013, when only CFA positive individuals were examined for mf, the community mf prevalence was calculated as: (b/a) _*_ (d/c) _*_ 100, where a = number of individuals in the community examined for CFA, b = number of those examined for CFA being positive, c = number of CFA positives examined for mf, and d = number of those examined for mf being positive [18]. In these surveys, the community mf GMI was calculated as antilog [(Σlog x +1)/(c/b) _*_ a] – 1, with x being the mf/ml blood and (c/b) _*_ a being the number of individuals examined (taking into account that sometimes not all CFA positive individuals were examined for mf).

Entomological indices for vector biting and transmission were calculated as described previously [13]. Briefly, the monthly biting rate (MBR, a measure of the number of mosquito bites per person in the month) was calculated as: (total mosquito catch _*_ days in month _*_ 3) / (number of catching nights _*_ number of light traps _*_ 2). The monthly transmission potential (MTP, a measure of the number of infective larvae to which a person is exposed in the month) was calculated as: (MBR _*_ total number of infective larvae seen in the dissections) / (number of mosquitoes dissected).

Categorical variables were compared statistically by chi-square test, whereas continuous variables were compared by t-test or one-way analysis of variance, as appropriate. P-values less than 0.05 were considered statistically significant.

## Results

### Tanga District communities

Results from the pre-MDA survey in Kirare village in 2004 are summarized in Table 1. High levels of LF infection and chronic disease were recorded, with 63.3% and 33.6% of individuals aged ≥10 years being positive for CFA and mf, respectively, 42.7% of males aged ≥15 years having hydrocele and 5.2% of individuals aged ≥15 years having elephantiasis.

**Table 1:** **Overview of the LF status in Kirare, as seen during the pre-MDA survey in September 2004**

**Characteristic**	
Registered population (≥10 years)	336
Microfilaraemia (≥10 years)	
Prevalence	33.6%
GMI^a^ among examined	8.8
GMI^a^ among positive	881
CFA^b^ prevalence (≥10 years)^c^	63.3%
Bm14 prevalence (≥10 years)^c^	85.7%
Hydrocele prevalence in males (≥15 years)^d^	42.7%
Elephantiasis prevalence in all (≥15 years)^d^	5.2%

Based on data from Mtambuuni and Mashine hamlets.

^a^Geometric mean intensity, in mf/ml blood.

^b^Circulating filarial antigens.

^c^Assessed in volunteers from mosquito collection houses (n =49).

^d^Assessed in 117 males (hydrocele) and 269 males and females (elephantiasis), respectively.

**Figure 2:**
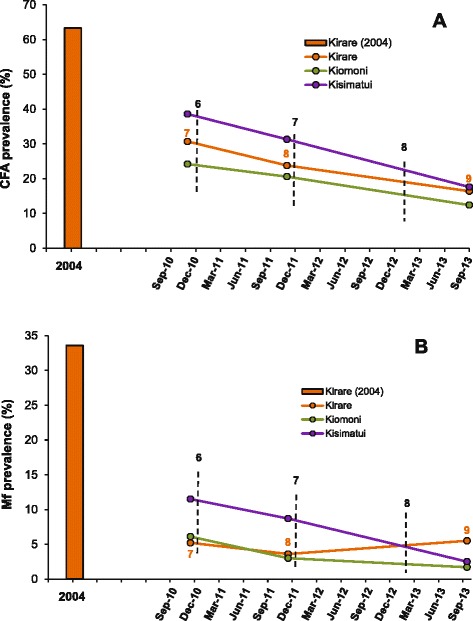
**Prevalence of circulating filarial antigens (A) and microfilaraemia (B) in the rural study communities in Tanga District during surveys 1 and 7–9.** Data are from individuals aged ≥10 years from two hamlets of Kirare (Mtambuuni, Mashine), one hamlet of Kiomoni (Mabavu) and one hamlet of Kisimatui (Majengo). Bar = prevalence in Kirare at the pre-MDA survey in 2004; Orange line = prevalence in Kirare in survey 7–9; Green line = prevalence in Kiomoni in survey 7–9; Blue line = prevalence in Kisimatui in survey 7–9. In the pre-MDA survey all individuals were examined for mf, and volunteers from mosquito collection houses only were examined for CFA by ELISA. In surveys 7–9 all individuals were first examined for CFA with ICT cards, and those positive were examined for mf. Vertical stippled lines indicate rounds of MDA.

The LF infection results from surveys 7, 8 and 9 in Kirare, Kiomoni and Kisimatui villages are shown in Table 2, and these results are compared to those from Kirare in 2004 in Figure 2. Both CFA and mf prevalences were considerably lower in the later surveys than in Kirare in 2004, and on average for the three villages reached 15.5% and 3.5%, respectively during survey 9 (decrease by 75.5% and 89.6%, respectively, compared to the baseline level in Kirare). The decrease in CFA prevalence between survey 7 and 9 was statistically significant in all three villages, and for the villages combined it was highly significant between each individual survey (p =0.009 between survey 7 and 8; p <0.001 between survey 8 and 9). The decrease in mf prevalence and mf GMI among all examined between survey 7 and 9 was also obvious for the three villages combined, although a slight increase was noted in Kirare between survey 8 and 9 (statistics cannot be computed due to the method of assessing these indices, see Methods).

**Table 2:** **Circulating filarial antigens and microfilariae in the rural study communities in Tanga District**

**Community** ^**a**^	**Time (number) of survey**	**Survey population**	**Circulating filarial antigen**			**Microfilariae**				
			**No. examined**	**No. positive (%)**	**p-value** ^**b**^	**No. examined** ^**c**^	**No. positive**	**Community mf prevalence** ^**c**^	**Mf GMI** ^**d**^ **for all examined**	**Mf GMI** **for positives**
Kirare	Nov-10 (7)	554	319	98 (30.7)	0.051	89	15	5.2	0.31	178.9
	Nov-11 (8)	554	311	74 (23.8)		60	9	3.6	0.20	180.8
	Sep-13 (9)	624	422	69 (16.4)	0.012	60	20	5.5	0.31	293.7
Kiomoni	Nov-10 (7)	394	293	71 (24.2)	0.323	68	17	6.1	0.44	423.1
	Nov-11 (8)	394	233	48 (20.6)		48	7	3.0	0.20	487.3
	Sep-13 (9)	451	338	42 (12.4)	0.008	36	5	1.7	0.10	282.2
Kisimatui	Nov-10 (7)	492	277	107 (38.6)	0.075	91	27	11.5	0.80	171.7
	Nov-11 (8)	492	259	81 (31.3)		61	17	8.7	0.46	74.5
	Sep-13 (9)	517	301	53 (17.6)	<0.001	42	6	2.5	0.13	107.7
All three combined	Nov-10 (7)	1440	889	276 (31.0)	0.009	248	59	7.4	0.49	225.0
	Nov-11 (8)	1440	803	203 (25.3)		169	33	4.9	0.28	141.6
	Sep-13 (9)	1592	1061	164 (15.5)	<0.001	138	31^e^	3.5	0.21	240.4

Results are from individuals aged ≥10 years during surveys 7, 8 and 9. Results from earlier surveys were given in [18].

^a^Two hamlets of Kirare (Mtambuuni, Mashine), one hamlet of Kiomoni (Mabavu) and one hamlet of Kisimatui (Majengo).

^b^For difference in prevalence (Pearson chi-square test).

^c^Only CFA positives were examined. See Methods for calculation.

^d^Geometric mean intensity, in mf/ml blood. See Methods for calculation.

^e^Eight females, 23 males (mean age: 37.1 years; range 10–73 years).

The chronic LF morbidity results from survey 9 (2013) are compared to those from the pre-MDA survey in Kirare (2004) in Table 3. It is remarkable that the prevalence of chronic signs for both hydrocele and elephantiasis in 2013 was less than half of that in 2004. For hydrocele, this decrease in prevalence was highly significant, both when comparing Kirare in 2004 to Kirare in 2013 and when comparing Kirare in 2004 to all three villages combined in 2013 (p <0.001 for both tests). For elephantiasis, the decrease was only significant for the second but not for the first of these comparisons (p =0.004 and p =0.11, respectively), most likely because of the low numbers of cases in the former. It is noteworthy in this respect that the mean age of those with hydrocele and elephantiasis did not differ significantly between 2004 and 2013 (p >0.05 for all tests).

**Table 3:** **Chronic LF morbidity in the rural study communities in Tanga District**

**Survey number (year) and community** ^**a**^	**Hydrocele (males only)**					**Elephantiasis (females and males)**				
	**No. examined**	**No. positive (%)**	**p-value** ^**b**^	**Mean age (range) of positives**	**p-value** ^**c**^	**No. examined**	**No. positive (%)**	**p-value** ^**a**^	**Mean age (range) of positives**	**p-value** ^**b**^
Survey 1 (2004)										
Kirare	117	50 (42.7)	-	50.2 (16–76)	-	269	14 (5.2)	-	51.1 (26–74)	-
Survey 9 (2013)										
Kirare	123	22 (17.9)	< 0.001	53.7 (16–90)	0.43	334	8 (2.4)	0.11	54.9 (41–84)	0.58
All 3 communities	304	44 (14.5)	< 0.001	53.8 (16–90)	0.31	808	14 (1.7)	0.004	54.9 (36–85)	0.52

Results are from individuals aged ≥15 years examined during the pre-MDA survey (2004) and after 8 rounds of MDA (2013).

^a^Two hamlets of Kirare (Mtambuuni, Mashine), one hamlet of Kiomoni (Mabavu) and one hamlet of Kisimatui (Majengo).

^b^Pearson chi-square test comparing prevalence in 2004 to prevalences in 2013.

^c^t-test comparing mean age in 2004 to mean ages in 2013.

**Figure 3:**
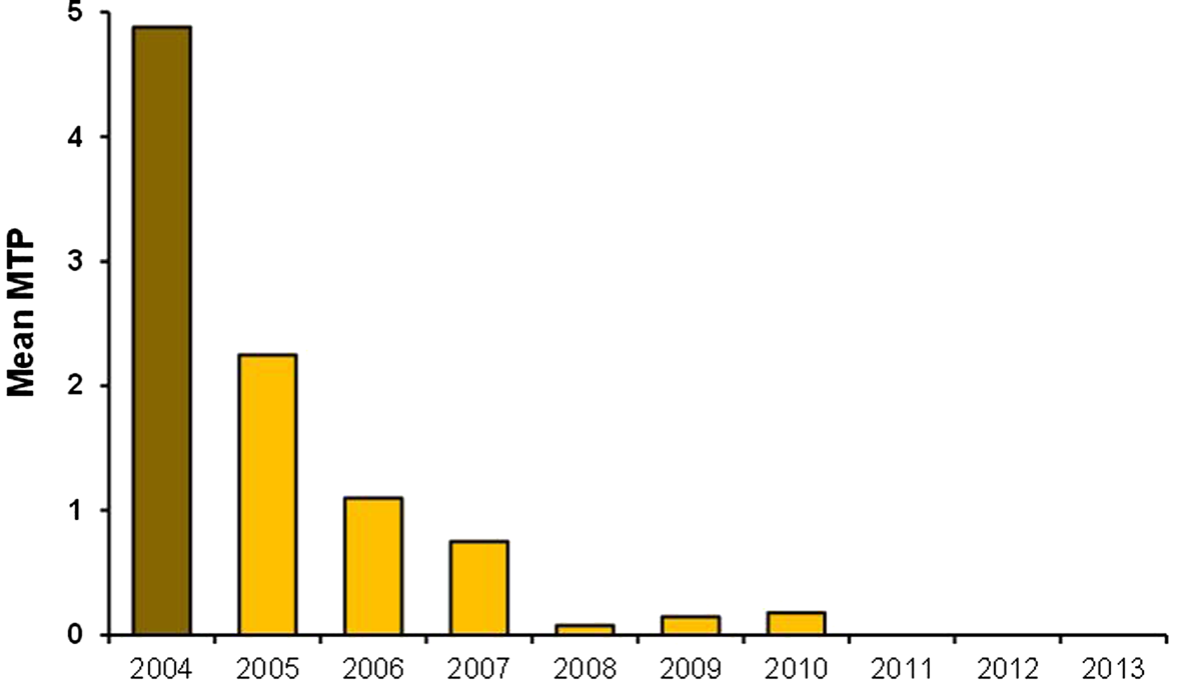
**Mean monthly transmission potential (MTP) for the main transmission months of May-August for the years of 2004–2013.** Only 2 months included for 2012 and 2013 (see Methods).

Results from the two months of vector and transmission surveillance in the post-MDA 7 and post MDA 8 periods are shown in Table 4, together with findings from the full-time pre-MDA and post-MDA 6 periods. When comparing results, it should be noted that not only the length of collection period differed, but also that the post-MDA 7 and 8 collections were made towards the end of the long rainy season when peak mosquito density was expected. The post-MDA 7 period thus had exceptionally low vector mosquito density, whereas the density was higher in the post-MDA 8 period. The previously reported [16,18] shift in vector composition from being predominantly anophelines during the pre-MDA period to being predominantly *Culex quinquefasciatus* in the later part of the study was still clearly seen. None of the dissected vector mosquitoes from post-MDA periods 7 or 8 were found to carry filarial infections. The mean MTP for the four main transmission months of May-August for the years 2004 to 2013 is shown in Figure 3 (only 2 months included for 2012 and 2013; see Methods) and indicates a dramatic decrease in transmission during the study period.

**Table 4:** **Vector mosquito catches from the 50 collection houses in Kirare, and the outcome of dissections**

**Period**	**Months of collection**	**No. collected (mean per month)**	**Mean monthly biting rate**	**No. dissected**	**No. infected** ^**a**^ **(% of dissected)**	**No. infective** ^**b**^ **(% of dissected)**	**Mean monthly transmission potential**
Pre-MDA (Nov-03 to Sep-04)	11						
*An. gambiae*		2335 (212.3)	49.39	1477	56 (3.8)	20 (1.35)	1.90
*An funestus*		3385 (207.7)	71.3	2080	94 (4.5)	48 (2.31)	3.47
*Cx. quinquefasciatus*		2626 (238.7)	53.7	1839	37 (2.0)	9 (0.49)	0.69
Total		8346 (758.7)	174.3	5396	187 (3.5)	11 (1.43)	6.08
Post-MDA 6 (Dec-10 to Nov-11)	12						
*An. gambiae*		416 (34.7)	7.3	387	0 (0.0)	0 (0.0)	0.00
*An funestus*		74 (6.2)	1.4	74	0 (0.0)	0 (0.0)	0.00
*Cx. quinquefasciatus*		8492 (707.7)	153.9	7252	2 (0.03)	1 (0.01)	0.05
Total		8982 (748.5)	161.8	7713	2 (0.03)	1 (0.01)	0.05
Post-MDA 7^c^	2						
*An. gambiae*		24 (12.0)	5.2	19	0	0	-
*An funestus*		26 (13.0)	5.7	8	0	0	-
*Cx. quinquefasciatus*		104 (52.0)	22.7	98	0	0	-
Total		154 (77.0)	33.6	125	0	0	-
Post-MDA 8^d^	2						
*An. gambiae*		246 (123.0)	53.6	226	0	0	-
*An funestus*		0 (0.0)	0.0	0	0	0	-
*Cx. quinquefasciatus*		1454 (727.0)	316.8	1306	0	0	-
Total		1700 (850.0)	370.4	1532	0	0	-

Mosquitoes were collected in all four hamlets of Kirare. Results shown are from the full pre-MDA and post-MDA 6 periods as well as from two months of collection during the peak mosquito seasons in 2012 and 2013. Results from the post-MDA 1–5 periods were given in [16,18].

^a^With any larval stage (L1, L2, L3) of *W. bancrofti*; ^b^With L3 larval stage of *W. bancrofti*.

^c^June/July, 2012 (42 sampling nights; 420 trap nights); ^d^May/June, 2013 (42 sampling nights; 420 trap nights).

The surveyed treatment coverages for MDA 7 and 8 in Tanga District were generally on the lower side. Thus, at the post-MDA 7 questionnaire survey in January 2012, 55.9% of 1432 interviewed community members ≥5 years indicated they had taken the drugs. At the post-MDA 8 questionnaire survey in February 2013, 50.5% of 1399 interviewed community members ≥5 years indicated they had taken the drugs. The reported treatment coverages from the programme for the entire Tanga District at these two MDAs were 51% and 46%, respectively.

Results from the questionnaire survey among the 32 mf positive individuals identified during survey 9 are shown in Table 5. A total of 29 individuals participated, of which most (86.2%) indicated they had stayed more than 5 years in the village. The great majority (89.7%) indicated they knew about the MDA programme and 82.8% expressed the opinion that it was important that everyone in the community should take the tablets. However, 23 (79.3%) admitted that they did not take the tablets last time because they were absent (13), tablets were not distributed (5), they were not informed about the distribution (3), they did not like the tablets (1) or they feared for side effects (1). Thus, 21 (91.3% of those who did not take the tablets) indicated that they had not been offered the tablets during last MDA, as an explanation for their non-compliance. All indicated they would take the tablets during next MDA round.

**Table 5:** **Questionnaire-based assessment of microfilaria positive individuals in the three rural study communities in Tanga District**

	**No. responses (%)**
*How long time have you lived in this village (n =29)?*	
1-5 years	3 (10.3)
> 5 years	25 (86.2)
No answer	1 (3.4)
*Have you heard about the MDA programme (n =29)?*	
Yes	26 (89.7)
No	3 (10.3)
*Did you take the MDA tablets in February 2013 (n =29)?*	
Yes	4 (13.8)
No	23 (79.3)
Don’t know	2 (6.9)
*Why did you not take the tablets (n =23)?*	
Was absent	13 (56.5)
Tablets not distributed	5 (21.7)
Not informed	3 (13.0)
Not like the tablets	1 (4.3)
Fear of side effects	1 (4.3)
*Did you take the tablets during previous MDAs (n =29)?*	
No	9 (31.0)
Yes, once	6 (20.7)
Yes, twice	8 (27.6)
Yes, more than twice	5 (17.2)
Don’t know	1 (3.4)
*Why not (n =9)?*	
Was absent	7 (77.8)
Tablets not distributed	1 (11.1)
Fear of side effects	1 (11.1)
*Will you take the tablets during next MDA (n =29)?*	
Yes	29 (100.0)
*Why will you take the tablets during next MDA (n =29)?*	
To protect me from getting LF	22 (75.9)
To prevent spread of LF in the village	5 (17.2)
Instructed by village leaders	1 (3.4)
Because many people take them	1 (3.4)

29 of the 31 individuals found mf positive during survey 9 (September 2013) in Kirare, Kiomoni and Kisimatui were interviewed (7 females, 22 males; mean age 39.4 years; age range 8–73 years).

### Tanga District schools

**Figure 4:**
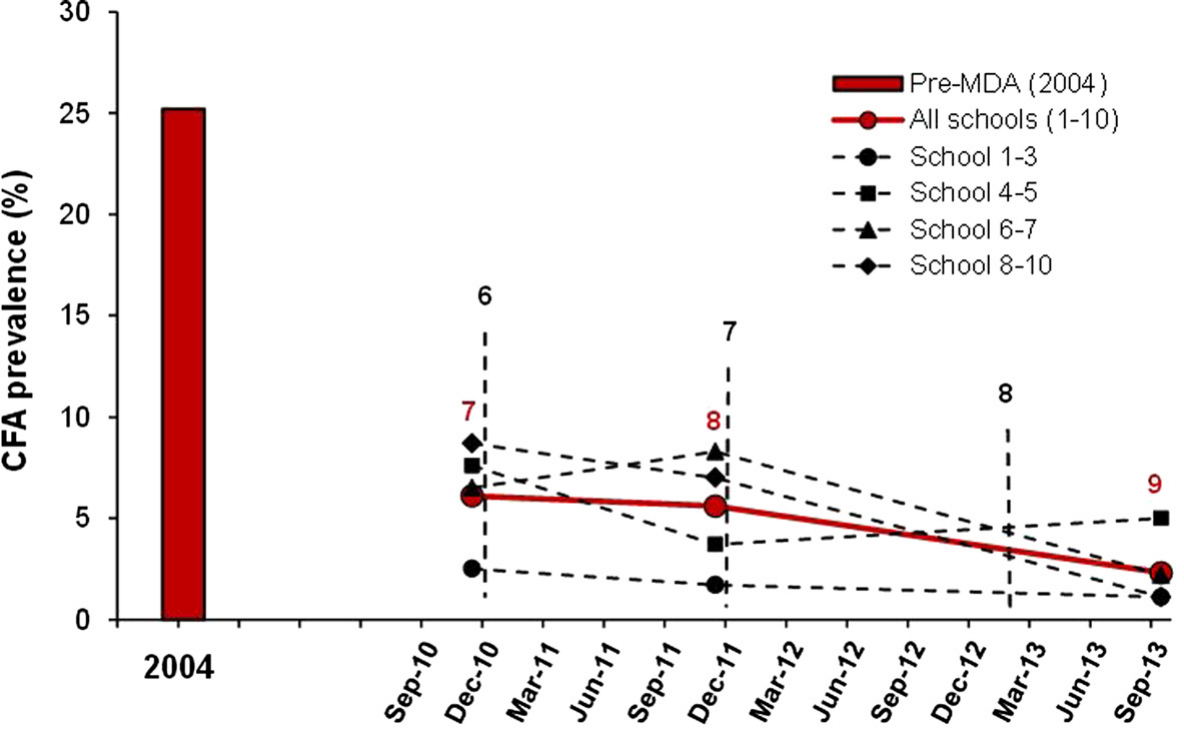
**Prevalence of circulating filarial antigens in Standard 1 pupils from 10 rural primary schools in Tanga District during surveys 1 and 7–9.** Bar = prevalence in all 10 schools combined at the pre-MDA survey in 2004. Thick line = prevalence for all 10 schools combined during surveys 7–9. Thin stippled lines = prevalence in school clusters according to their location to the south (Kirare, Mapojoni and Marungu schools; no. 1–3), north (Kiomoni and Mafuriko schools; no. 4–5), close west (Maweni and Kange schools; no. 6–7) and more distant west (Pongwe, Kigandini and Ziwani schools; no. 8–10) of Tanga city during survey 7–9. Vertical stippled lines indicate rounds of MDA.

The new intakes of Standard 1 pupils from the 10 study schools were examined for CFA shortly after starting school. The results from surveys 7, 8 and 9 are shown in Table 6, and these results are compared to those from 2004 in Figure 4. The average CFA prevalence for the 10 schools combined decreased progressively from year to year, and reached 2.3% during survey 9 (from a pre-MDA prevalence of 25.2%; decrease by 90.9%). The decrease in CFA prevalence between survey 7 and 8 was not statistically significant (p =0.65), whereas between survey 8 and 9 it was highly significant (p <0.001). Similar trends were seen in the four school clusters although with some fluctuations due to the low number of CFA positive pupils.

**Table 6:** **Circulating filarial antigen (CFA) in Standard 1 pupils from 10 rural primary schools in Tanga District**

**Time (number) of survey**	**No. pupils registered**	**No. examined (% of registered)**	**No. girls/boys (ratio)** ^**a**^	**Mean age in years (range)** ^**a**^	**No. positive for CFA (% of examined)**	**p-value for change in CFA prevalence from previous year** ^**b**^
Nov-10 (7)	966	831 (86.0)	404/427 (0.95)	7.5 (6–12)	51 (6.1)	0.80
Dec-11 (8)	943	889 (94.3)	421/468 (0.92)	7.5 (6–11)	50 (5.6)	0.65
Sep-13 (9)	1124	990 (88.1)	492/488 (1.01)	7.6 (6–12)	23 (2.3)	< 0.001

Each year, the new intake of Standard 1 pupils in the 10 schools (Kiomoni, Mafuriko, Marungu, Kirare, Mapojoni, Pongwe, Kigandini, Maweni, Ziwani and Kange) was examined. Results from earlier surveys were given in [17,18].

^a^For examined pupils.

^b^Pearsons chi-square test.

The post-MDA questionnaires among Standard 1 pupils in the 10 schools indicated that only 18.8% had taken the treatment in MDA 7 (among 845 pupils interviewed in January 2012) whereas 44.9% had taken the treatment in MDA 8 (among 770 pupils interviewed in February 2013).

### Other districts of Tanga Region

**Figure 5:**
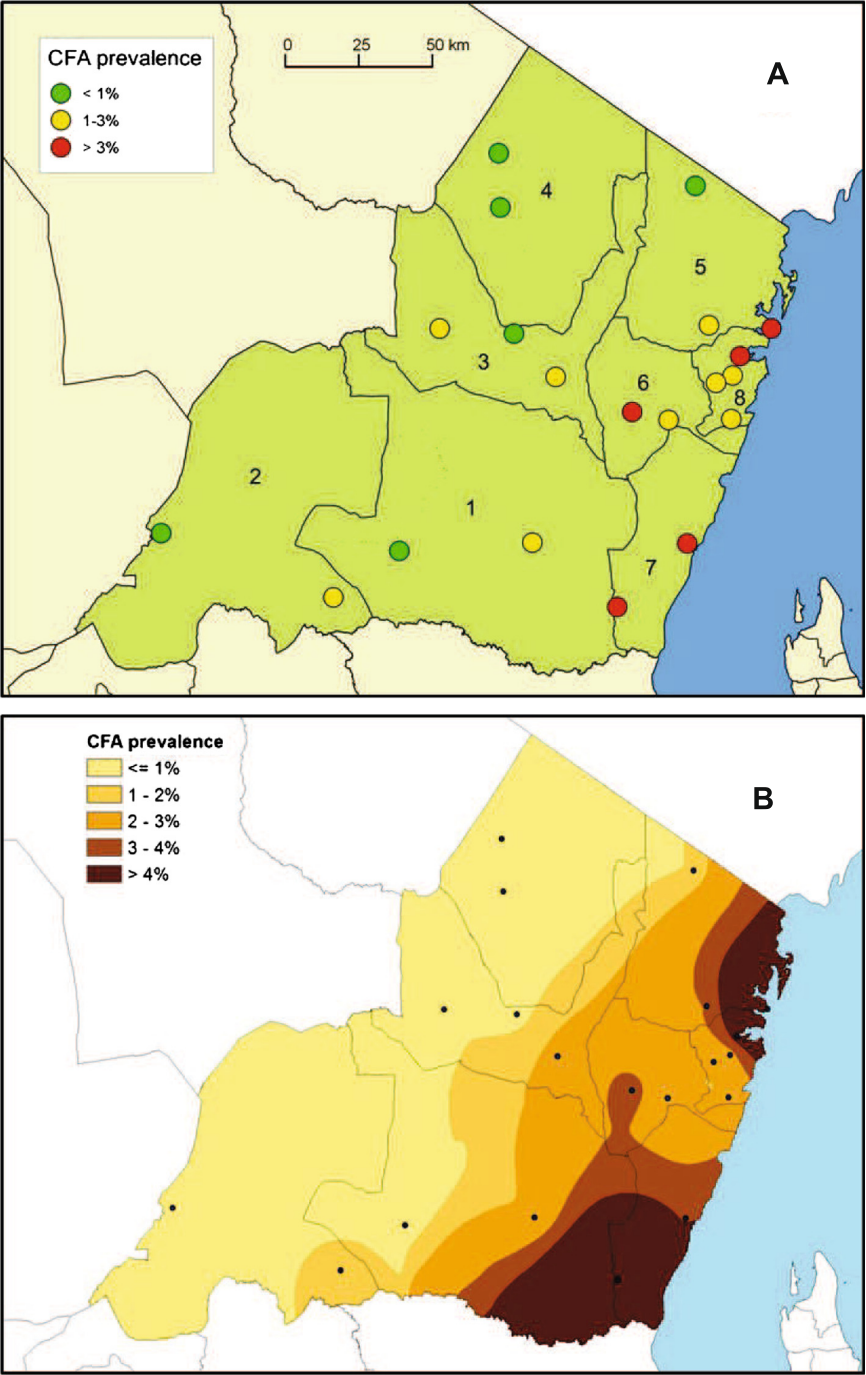
**Map of Tanga Region showing circulating filarial antigen (CFA) prevalence in primary school children in 2013. A** = measured prevalence levels at the survey sites. **B** = Prevalence isolines prepared by Bayesian kriging on the empirical data. Numbers indicate districts: 1 = Handeni, 2 = Kilindi, 3 = Korogwe, 4 = Lushoto, 5 = Mkinga, 6 = Muheza, 7 = Pangani, 8 = Tanga.

Results from the spot check surveys for CFA in the 16 schools in the other 7 districts of Tanga Region are shown in Table 7. CFA prevalences were low in the inland districts of Handeni, Kilindi, and Lushoto (average of 0.5% for the 1204 examined pupils at the 6 sites), intermediate in Korogwe and Muheza (average of 1.8% for the 1004 examined pupils at the 5 sites) and highest in the coastal districts of Mkinga and Pangani (average of 5.5% for the 998 pupils examined at the 5 sites), and the difference in prevalence between these three groups was statistically significant (p <0.001). Highest CFA prevalence (11.0%) was recorded in Mkalamo School in Pangani District. The difference in pattern of CFA prevalence in schoolchildren between inland and coastal sites is also obvious from Figure 5A (in which results from the 4 school clusters in Tanga District have also been included). Kriging on the empirical data from the 16 schools plus the four school clusters in Tanga district was used to prepare and show CFA-prevalence iso-lines on the regional map (Figure 5B). The map indicates that all of Kilindi and Lushoto districts and most of Korogwe District are located on the lower site of the 2% CFA-prevalence iso-line.

**Table 7:** **School surveys for circulating filarial antigen in the other seven districts of Tanga Region**

**District**	**School**	**Classes included**	**No. examined**	**Mean age in years (range)**	**Female: male ratio**	**No. positive (%)**
Handeni (1)	Madebe	1-6	200	9.9 (7–16)	1.1	1 (0.5)
	Komkonga	1-2	199	8.2 (7–11)	1.4	2 (1.0)
Kilindi (2)	Songe	1-2	202	7.8 (6–12)	1.5	0 (0.0)
	Lukole/Negero	1-6	202	10.0 (6–16)	1.3	3 (1.5)
Korogwe (3)	Kwamndolwa	1-3	203	8.5 (7–11)	1.3	4 (2.0)
	Vuluni	1-6	200	10.2 (6–15)	1.1	1 (0.5)
	Mkalamo	1-7	200	9.9 (7–14)	1.2	2 (1.0)
Lushoto (4)	Mwangoi	1-3	200	9.2 (6–14)	1.2	0 (0.0)
	Mnazi	1-3	201	9.6 (6–15)	1.2	0 (0.0)
Mkinga (5)	Kwale	1-6	199	9.6 (7–15)	1.3	19 (9.5)
	Gombero	1-6	200	9.9 (5–15)	1.0	6 (3.0)
	Mwakijembe	1-3	202	9.3 (7–14)	1.2	1 (0.5)
Muheza (6)	Mkuzi	1-3	200	9.7 (7–13)	1.0	3 (1.5)
	Mamboleo	1-3	201	9.6 (6–14)	1.0	8 (4.0)
Pangani (7)	Kipumbwi	1-2	197	7.8 (7–12)	1.0	7 (3.6)
	Mkalamo	1-3	200	8.4 (7–14)	1.0	22 (11.0)
Total	-	-	3206	9.2 (5–16)	1.2	79 (2.5)

Examinations started with Standard 1 pupils and thereafter continued progressively to higher classes until the required number had been examined.

**Figure 6:**
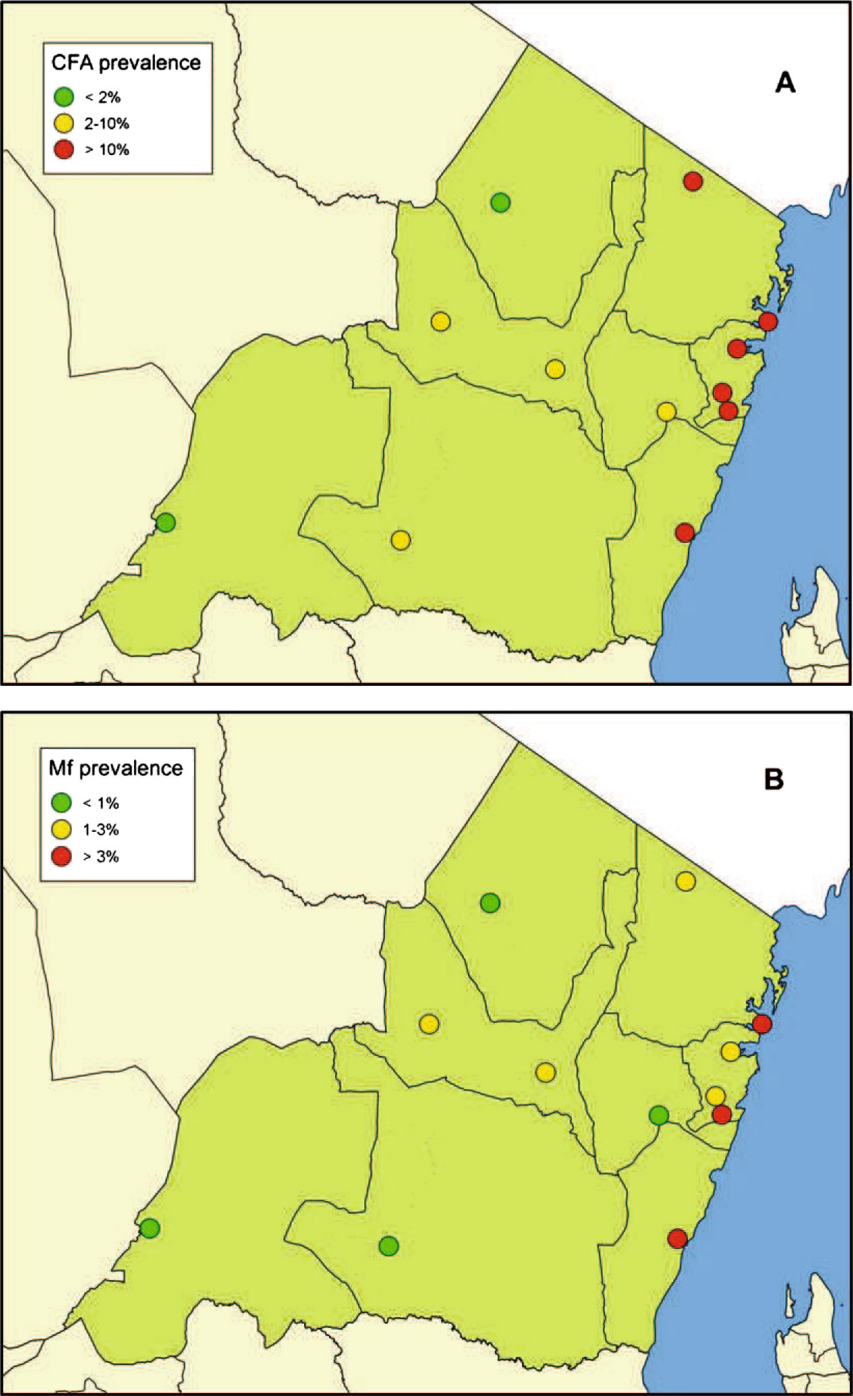
**Map of Tanga Region showing community prevalence of circulating filarial antigens (A) and microfilariaemia (B) in 2013.** Volunteers aged ≥10 years were first examined for circulating filarial antigens (CFA) with ICT cards and those positive were examined for microfilariae (Mf).

Results from the spot check surveys in the 9 communities in the other 7 districts of Tanga Region are shown in Table 8. As expected, the community CFA prevalences were higher than those of the schoolchildren, but a similar pattern was seen. The most inland districts of Handeni, Kilindi and Lushoto had the lowest prevalences (average of 1.1% for the 560 individuals at the 3 sites), Korogwe and Muheza districts had intermediate prevalences (average of 7.2% for the 609 individuals at the 3 sites), and the coastal districts of Mkinga and Pangani had highest prevalences (average of 20.0% for the 596 individuals tested). The difference in prevalence between the three groups was statistically significant (p <0.001). Microfilariae were only detected in the districts with the highest CFA prevalences (Pangani, Mkinga, Korogwe). These patterns are also obvious from Figure 6, in which results from the three study communities in Tanga District have also been included.

**Table 8:** **Community surveys for LF infection in the other seven districts of Tanga Region**

**District**	**Village**	**Circulating filarial antigen**				**Microfilarae**		
		**No. examined**	**Mean age in years (range)**	**Female: male ratio**	**No. positive (%)**	**No. examined** ^**a**^	**No. positive**	**Community mf prevalence in %** ^**b**^
Handeni (1)	Madebe	181	30.7 (10–80)	0.8	4 (2.2)	2	0	0.0
Kilindi (2)	Songe	180	23.8 (10–100)	0.5	0 (0.0)	0	0	0.0
Korogwe (3)	Kwamdolwa	207	40.2 (10–86)	1.2	16 (7.7)	15	2	1.0
Korogwe (3)	Mkalamo	222	35.1 (15–94)	0.6	22 (9.9)	17	4	2.3
Lushoto (4)	Mwangoi	199	34.9 (10–90)	1.1	2 (1.0)	2	0	0.0
Mkinga (5)	Kwale	202	37.0 (10–100)	0.8	36 (17.8)	32	8	4.5
Mkinga (5)	Mwakijembe	200	42.5 (16–100)	0.6	35 (17.5)	23	4	3.0
Muheza (6)	Mkuzi	180	27.2 (10–84)	0.9	6 (3.3)	2	0	0.0
Pangani (7)	Kipumbwi	194	27.4 (10–90)	0.4	48 (24.7)	27	6	5.5
Total	-	1765	33.5 (10–100)	0.7	169 (9.6)	120	24^c^	1.9

Volunteers aged ≥10 years were examined.

^a^Only CFA positives were examined.

^b^See Methods for calculation.

^c^Five females, 19 males (mean age: 34.8 years; range 11–70 years); GMI =179.5 mf/ml blood.

The findings from clinical examination of community members reflected the LF infection patterns. Thus, the prevalence of hydrocele was significantly higher in coastal Pangani and Mkinga districts (10.2% for 332 examined males) than in the more inland Handeni, Kilindi, Lushoto, Korogwe and Muheza districts (3.1% for 522 examined males; p <0.001), and the prevalence of elephantiasis was significantly higher in the first two districts (3.8% for 494 examined individuals) than in the later five districts (0.3% for 930 examined individuals; p <0.001).

## Discussion

Since launching of the LF control programme in Tanga Region in 2004, the present project has monitored the effect of MDA on LF infection and transmission in rural areas of Tanga District through repeated human cross sectional surveys in communities and schools and continuous vector and transmission surveillance [16-18]. As infection and transmission indices had reached relatively low levels, and for logistical reasons, most activities were suspended in 2012. In 2013, the same study communities and schools in Tanga District were re-examined. In addition, it was decided in 2013 to carry out spot check surveys in communities and schools in the other 7 districts of Tanga Region, to assess the overall regional LF status after 8 rounds of MDA.

In the longitudinal study in Tanga District, the human infection burden continued to decrease from one survey to the next. After 8 rounds of MDA in 2013, the CFA and mf prevalences in the combined study communities were reduced by 75.5% and 89.6%, respectively, compared to the baseline level in Kirare, and in the 10 study schools, the CFA prevalence on average was reduced by 90.9% compared to baseline. Continued decrease in these indices was also obvious in the later part of the study in both communities and schools, although it was less evident between survey 7 and 8 (where only one MDA was administered) than between survey 8 and 9 (where 2 MDAs were administered). The mf GMI among the examined individuals in the three communities was reduced even more (by 97.6% compared to baseline in Kirare), thus indicating a considerable reduction in number of mf being available for onward transmission by vector mosquitoes. On the other hand, the mf GMI among individuals who were still mf positive remained very high, suggesting that some of these more systematically had missed the annual MDAs (systematic non-compliers) and served as important sources of mf for continued transmission.

Two issues are important to consider when evaluating this positive reduction in community and school LF infection burdens. First, the prevalence levels for both mf and CFA in 2013 were still well above those recommended for stopping MDA (1% and 2%, respectively) [5,22]. Second, although there can be little doubt that the major cause of the decrease was the MDAs, other factors contributed. Bed nets were rarely used in the beginning of the study in 2004 [16] but gradually became more common, and ITNs were distributed free of charge to all households in Tanga Region in 2011. Moreover, there has been a major change in vector mosquito density and species composition during the period of the study, as previously reported in detail [16,18,23], with a considerable decline in density of the important *Anopheles gambiae* and *An. funestus* vectors, and with the few remaining *An. gambiae* mainly being of the less anthropophilic *An. arabiensis* sibling species [24]. It is still unclear what exactly has caused this change, but climatic and other environmental changes, as well as the increased use of ITNs, have undoubtedly played important roles.

Other studies have assessed the effect of MDA on LF infection, especially in areas where control programmes have used the DEC/albendazole combination (e.g. [25-29]). DEC cannot be used for MDA in most countries of sub-Saharan Africa, because of the risk of severe adverse reactions in individuals with concomitant *O. volvulus* infection, and is substituted by ivermectin in these areas. Successful elimination of LF with the ivermectin/ albendazole combination was recently reported from Togo, which however had very low pre-MDA LF endemicity level [30,31]. Programmes in Sierre Leone [32] and Nigeria [33,34], partly building on existing and effective Community Directed Intervention programmes for control of onchocerciasis, and with low to moderate pre-MDA levels of LF endemicity and remarkably high treatment coverages, have also shown good progress with this combination. The LF elimination programme in Zanzibar similarly reported a marked drop in microfilaraemia after five rounds of ivermectin/albendazole MDA with intensive community mobilization and high treatment coverages [35]. The combination of very high LF endemicity at baseline and moderate treatment coverages is probably an important reason for the relatively high human infection level observed after 8 rounds of MDA in the coastal areas in the present study.

The aim of MDAs is to reduce the mf load in the human population to prevent infection of vectors and thereby ultimately to interrupt transmission [4,5]. The sharp decline observed in transmission in Kirare after the first MDAs, should most likely primarily be attributed to the effect of the treatment, but – as mentioned earlier - increased bed net use and changes in the vector population also played a role. The fact that no infective vector mosquitoes were detected after MDA 7 does not necessarily mean that transmission had been completely interrupted, − only that not enough mosquitoes were caught and examined for infective larvae. This is a major challenge in LF transmission surveillance, that trapping of enormous numbers of mosquitoes is necessary to detect infective ones when transmission level is low [36]. The recent increase in importance of *Culex quinquefasciatus* as LF vectors in the study area suggest that use of gravid traps [37], which tend to give a better yield with this species, might be a better option for entomological assessment of LF transmission in the future.

The marked reduction in chronic LF morbidity burden observed in the longitudinal community study, with prevalence of hydrocele and elephantiasis after 8 rounds of MDA being less than half of what it was at baseline, was unexpected. A positive effect of MDAs with the ivermectin/albendazole combination on lymphedema size was previously also reported from the Tanzanian NLFEP [7]. A number of possible explanations can be suggested, which alone or in combination may have contributed to this. First, the lowered transmission intensity and/or infection burden with increasing number of MDAs may have reduced or delayed development of new cases. Secondly, there could have been a direct, perhaps anti-inflammatory, effect of treatment on the chronic morbidity, although evidence for such properties of ivermectin or albendazole is meagre [38]. Thirdly, surgical removal of hydroceles, although not commonly performed in the area due to high cost and limited hospital capacity, may have reduced the number of hydroceles. Fourthly, better hygiene due to general improved standard of living (better availability of e.g. water, soap, shoes and antibiotics) may have contributed to reduce the frequency of superficial secondary bacterial/fungal infections which often trigger and aggravate lymphedema and elephantiasis formation [2,38]. Lastly, a number of incidents during field work suggested that some individuals with chronic morbidity in the later part of the study hid themselves to avoid examination, as previous examinations had not provided alleviation of their disease symptoms. Several cross-sectional follow-up studies in the past have suggested that DEC might have a positive effect on lymphedema and hydrocele, but cohort studies following individual patients over a period of time have not been able to confirm this [39,40].

During MDA 7 and 8, the reported treatment coverages for Tanga District as well as the surveyed treatment coverages in the study communities and schools were on the lower side. With a range of 46-56%, these were markedly lower than the minimum of 65% required for reaching LF transmission interruption within reasonable time [5]. Treatment coverages moreover appeared to have declined when compared with previous years [16,18]. To attain and maintain high treatment coverage has been a commonly reported challenge for LF control programmes, both in Tanzania [41] and elsewhere [42]. The relatively high mf GMI among individuals who were still mf positive suggested that many of these had been poorly covered by the MDAs. This was confirmed in the interviews with the mf positive individuals, which moreover indicated that major reasons for not being treated were: 1) individuals had been away during distribution; 2) the tablets had not been distributed; or 3) individuals had not been informed about the distribution. Similar findings were reported from a recent larger study on MDA drug uptake in other parts of Tanzania, which concluded that although some people for various reasons refuse to take the tablets, compliance depended more on provider related factors than on individual perceptions and practices [41]. The present study also indicated that bed nets gradually gained more popularity in the populations during the period. A particularly marked increase in bed net coverage was - not surprisingly - observed immediately after the free distribution of ITNs in 2011. However, this rapidly dropped to more moderate level the following year, when it was also commonly observed in the surroundings that many nets were used for other purposes than covering human beds (like protection of chicken, seedlings and tomato plants, for fishing, and for collection of plastic bottles for recycling). Possibly some of these nets had been damaged and therefore could no longer serve their original purpose.

The spot-check surveys in 2013 showed considerable differences in LF infection and disease burden between the different parts of Tanga Region. Highest prevalences of CFA and mf were seen in the coastal areas, whereas there were lower prevalences in the more inland areas and particularly little infection in the highlands in the north-western and south-western parts of the region. A similar pattern, although with much higher prevalences, was reported in the 1950’s by Jordan [43]. It is likely that this distinct pattern largely is shaped by environmental conditions affecting the vectors, such as altitude, temperature, rainfall and humidity. Baseline data had originally been collected by the NLFEP from various sites in Tanga Region, but these unfortunately had irregularities that made them of little value for comparison to the data from the present study. The three MDAs with ivermectin applied for onchocerciasis control in highland areas of the Usambara Mountains before start of the LF control may potentially also have had an impact on LF infection in co-endemicity areas, as reported from elsewhere [44,45]. When compared to the rest of the coastal area, Tanga District was noted to have particularly low CFA prevalences in schoolchildren. It is tempting to speculate that control activities had been more intense near Tanga city, which accommodates the regional headquarters for the LF control programme, than in the districts to the North (Mkinga) and South (Pangani), although this was not immediately reflected in the reported district treatment coverages. A recent study on urban LF in Tanga city also indicated that a considerable decrease in transmission had occurred in recent years [46]. The spot check data from the present study will be a useful reference for future assessment of regional progress in LF control. They moreover provide a background for considering a face-out of MDA activities in the two districts with little LF (Lushoto and Kilindi) by following WHO guidelines [5,22], which could perhaps also release resources for more concentrated control efforts in the more affected districts.

Overall, the study showed that LF still is widespread in many parts of Tanga Region after 8 rounds of MDA, in particular in the coastal areas. This calls for intensified control, to effectively eliminate transmission. In this respect, some lessons learned from the present study might be useful. First, the MDA activity needs to be strengthened to ensure higher treatment coverage. This includes raising the population’s awareness of LF and interest in the MDA activity, and not least to ensure that all those who are already convinced and prepared to participate in the treatment campaigns also get the opportunity [15,41]. This will probably mean that more time has to be spent on the drug distribution activity and on informing and engaging the populations, but the alternative will be that treatment coverage remains too low. More frequent treatment (e.g. half yearly) could also be considered for the high endemicity areas, and perhaps even the use of DEC/albendazole instead of the ivermectin/albendazole combination, now that *O. volvulus* transmission has been brought close to elimination in the previous onchocerciasis endemic highland areas. In respect to the latter option it is noteworthy that DEC/albendazole is used for LF control in the nearby coastal districts of Kenya [47].

Second, there is a need for further advocacy to strengthen bed net usage. There has been increasing use of bed nets in the area during the past decade. However, the very high bed net coverage noted in 2011 was somehow inflated, due to the free household distribution of ITNs, and it declined to some extent afterwards. Bed net use can be an important supportive measure for LF control [48-50], but it is necessary to invest efforts in motivating and engaging people in correct usage of the nets to give optimal health benefits. Otherwise many nets will be transformed into other useful appliances during the seasons with low mosquito densities. Third, there should be more attention to the fact that males are the main LF infection carriers and sufferers, especially after initiation of control. In the present 2013 surveys, 74.5% and 79.2% of the remaining mf positive individuals in Tanga District and in the other districts of the region, respectively, were males (thus contributing most to transmission), and 87.9% and 84.7% of those with chronic LF disease, respectively, were males (due to the high frequency of hydrocele). This could be approached e.g. by having a more deliberate male focus in LF health messages, by making hydrocelectomies more accessible for the less wealthy male population, and by disseminating LF messages not only from health centres (which are often congested with females and small children) and during public meetings, but also from sites where men feel more comfortable and relaxed like bars, churches and mosques.

## Conclusions

The positive downward trend in LF transmission and human infection previously reported from the highly endemic Tanga District continued. However, LF was still widespread in many parts of Tanga Region after 8 rounds of MDA, in particular in the coastal areas to the north and south of Tanga city. This calls for intensified control in order to convincingly reach human infection levels below which transmission can no longer be maintained. In particular this should include strengthened efforts to increase the MDA treatment coverage (to at least ensure that everybody are offered the treatment), further support to and advocacy for use of bed nets (to prevent human infection and onward transmission), and more male focus in health dissemination (as males are the main reservoir of mf for infection of vectors). The particular low LF prevalences observed in some of the inland districts moreover suggest that MDA in these could be stepped down after rigorous assessment, to provide more resources for upscale of control activities in the coastal areas. Monitoring and evaluation should continue to guide the programme to ensure that the current major achievements will ultimately lead to successful LF elimination.

## References

[1] Simonsen PE, Fischer PU, Hoerauf A, Weil GJ, Farrar J, Hotez PJ, Junghanss T, Kang G, Lalloo D, White NJ (2014). The Filariases. Manson’s Tropical Diseases.

[2] WHO: *Lymphatic Filariasis: Managing Morbidity and Preventing Disability.* Geneva: World Health Organization; 2013.

[3] Ottesen EA (2006). Lymphatic filariasis: treatment, control and elimination. Adv Parasitol.

[4] WHO (2010). Global Programme to Eliminate Lymphatic Filariasis. Progress report 2000–2009 and strategic plan 2010–2020.

[5] WHO: *Monitoring and Epidemiological Assessment of Mass Drug Administration in the Global Programme to Eliminate Lymphatic Filariasis: A Manual for National Elimination Programmes.* Geneva: World Health Organization; 2011.

[6] Hotez PJ, Kamath A (2009). Neglected tropical diseases in Sub-Saharan Africa: review of their prevalence, distribution and disease burden. PLoS Negl Trop Dis.

[7] Malecela MN, Lazarus W, Mwingira U, Mwakitalu E, Makene C, Kabali C, Mackenzie C (2009). Eliminating LF: a progress report from Tanzania. J Lymphol.

[8] Minjas JN, Kihamia CN, Mwaluko GMP, Kilama WL, Mandara MP, Murru M, Macpherson CNL (1991). Bancroftian filariasis. Health and disease in Tanzania.

[9] McMahon JE, Magayuka SA, Kolstrup N, Mosha FW, Bushrod FM, Abaru DE, Bryan JH (1981). Studies on the transmission and prevalence of bancroftian filariasis in four coastal villages of Tanzania. Ann Trop Med Parasitol.

[10] Meyrowitsch DW, Simonsen PE, Makunde WH (1995). Bancroftian filariasis: analysis of infection and disease in five endemic communities of north-eastern Tanzania. Ann Trop Med Parasitol.

[11] Simonsen PE, Meyrowitsch DW, Makunde WH, Magnussen P (1995). Bancrotian filariasis: The pattern of microfilaraemia and clinical manifestations in three endemic communities of Northeastern Tanzania. Acta Trop.

[12] Simonsen PE, Meyrowitsch DW, Jaoko WG, Malecela MN, Mukoko D, Pedersen EM, Ouma JH, Rwegoshora RT, Masese NN, Magnussen P, Estambale BBA, Michael E (2002). Bancroftian filariasis infection, disease, and specific antibody response patterns in a high and a low endemicity community in East Africa. Am J Trop Med Hyg.

[13] Rwegoshora RT, Pedersen EM, Mukoko DA, Meyrowitsch DW, Masese N, Malecela-Lazaro MN, Ouma JH, Michael E, Simonsen PE (2005). Bancroftian filariasis: patterns of vector abundance and transmission in two East African communities with different levels of endemicity. Ann Trop Med Parasitol.

[14] CDI Study Group (2010). Community-directed interventions for priority health problems in Africa: results of a multi-country study. Bull Wld Health Org.

[15] Kisoka WL, Tersbøl BP, Meyrowitsch DW, Simonsen PE, Mushi DL: **Community members’ perceptions of mass drug administration for control of lymphatic filariasis in rural and urban Tanzania.** *J Biosocial Sci* 2014, In press. 10.1017/S0021932015000024 PMC4668335 25790081

[16] Simonsen PE, Pedersen EM, Rwegoshora RT, Malecela MN, Derua YA, Magesa SM (2010). Lymphatic filariasis control in Tanzania: Effect of repeated mass drug administration with ivermectin and albendazole on infection and transmission. PLoS Negl Trop Dis.

[17] Simonsen PE, Magesa SM, Derua YA, Rwegoshora RT, Malecela MN, Pedersen EM (2011). Monitoring lymphatic filariasis control in Tanzania: effect of repeated mass drug administration on circulating filarial antigen prevalence in young schoolchildren. Int Health.

[18] Simonsen PE, Derua YA, Kisinza WN, Magesa SM, Malecela MN, Pedersen EM (2013). Lymphatic filariasis control in Tanzania: effect of six rounds of mass drug administration with ivermectin and albendazole on infection and transmission. BMC Infect Dis.

[19] McMahon JE, Marshall TF, Vaughan JP, Abaru DE (1979). Bancroftian filariasis: a comparison of microfilariae counting techniques using counting chamber, standard slide and membrane (nuclepore) filtration. Ann Trop Med Parasitol.

[20] Pilz J, Spöck G (2007). Why do we need and how should we implement Bayesian kriging methods?. Stoch Environ Res Risk Assess.

[21] Simonsen PE, Niemann L, Meyrowitsch DW (1997). *Wuchereria bancrofti* in Tanzania: microfilarial periodicity and effect of blood sampling time on microfilarial intensities. Trop Med Int Health.

[22] Chu BK, Deming M, Biritwum NK, Dorkenoo AM, El-Setouhy M, Fischer PU, Gass K, de Pena MG, Mercado-Hernandez L, Kyelem D, Lammie PJ, Flueckiger RM, Mwingira UJ, Noordin R, Owusu IO, Ottesen EA, Pavluck A, Pilotte N, Rao BU, Samarasekera D, Schmaedick MA, Settinayake S, Simonsen PE, Supali T, Taleo F, Torres M, Weil GJ, Won KY (2013). Transmission Assessment Surveys (TAS) to define endpoints for lymphatic filariasis mass drug administration: a multicenter evaluation. PLoS Negl Trop Dis.

[23] Meyrowitsch DW, Pedersen EM, Alifrangis M, Scheike TH, Malecela MN, Magesa SM, Derua YA, Rwegoshora RT, Michael E, Simonsen PE (2011). Is the current decline in malaria burden in sub-Saharan Africa due to a decrease in vector population?. Malar J.

[24] Derua YA, Alifrangis M, Hosea KM, Meyrowitsch DW, Magesa SM, Pedersen EM, Simonsen PE (2012). Change in composition of the *Anopheles gambiae* complex and its possible implications for the transmission of malaria and lymphatic filariasis in north-eastern Tanzania. Malar J.

[25] Ramzy RMR, El Setouhy M, Helmy H, Ahmed ES, Elaziz KMA, Farid HA, Shannon WD, Weil GJ (2006). Effect of yearly mass drug administration with diethylcarbamazine and albendazole on bancroftian filariasis in Egypt: A comprehensive assessment. Lancet.

[26] Liang JL, King JD, Ichimori K, Handzel T, Pa’au M, Lammie PJ (2008). Impact of five annual rounds of mass drug administration with diethylcarbamazine and albendazole on *Wuchereria bancrofti* infection in American Samoa. Am J Trop Med Hyg.

[27] Weil GJ, Kastens W, Susapu M, Laney SJ, Williams SA, King CL, Kazura JW, Bockarie MJ (2008). The impact of repeated rounds of mass drug administration with diethylcarbamazine plus albendazole on bancroftian filariasis in Papua New Guinea. PLoS Negl Trop Dis.

[28] Ramaiah KD, Vanamail P, Yuvaraj J, Das PK (2011). Effect of annual mass administration of diethylbarbamazine and albendazole on bancroftian filariasis in five villages in South India. Trans R Soc Trop Med Hyg.

[29] Oscar R, Lemoine JF, Direny AN, Desir L, de Rochars VEMB, Poirier MJP, Varghese A, Obidegwu I, Lammie PJ, Streit TG, Milord MD (2014). Haiti National Program for the Elimination of Lymphatic Filariasis – A model of success in the face of adversity. PLoS Negl Trop Dis.

[30] Sodahlon YK, Dorkenoo AM, Morgah K, Nabiliou K, Agbo K, Miller R, Datagni M, Seim A, Mathieu E (2013). A success story: Togo is moving toward becoming the first sub-Saharan African nation to eliminate lymphatic filariasis through mass drug administration and countrywide morbidity alleviation. PLoS Negl Trop Dis.

[31] Budge PJ, Dorkenoo AM, Sodahlon YK, Fasuyi OB, Mathieu E (2014). Ongoing surveillance for lymphatic filariasis in Togo: assessment of alternatives and nationwide reassessment of transmission status. Am J Trop Med Hyg.

[32] Koroma JB, Sesay S, Sonnie M, Hodges MH, Sahr F, Zhang Y, Bockarie MJ (2013). Impact of three rounds of mass drug administration on lymphatic filariasis in areas previously treated for onchocerciasis in Sierre Leone. PLoS Negl Trop Dis.

[33] Richards FO, Eigege A, Miri ES, Kal A, Umaru J, Pam D, Rakers LJ, Sambo Y, Danboyi J, Ibrahim B, Adelamo SE, Ogah G, Goshit D, Oyenekan OK, Mathieu E, Withers PC, Saka YA, Jiya J, Hopkins DR (2011). Epidemiological and entomological evaluations after six years or more of mass drug administration for lymphatic filariasis elimination in Nigeria. PLoS Negl Trop Dis.

[34] King JD, Eigege A, Umaru J, Jip N, Miri E, Jiya J, Alphonsus KM, Sambo Y, Graves P, Richards F (2012). Evidence for stopping mass drug administration for lymphatic filariasis in some, but not all local government areas of Plateau and Nasarawa States, Nigeria. Am J Trop Med Hyg.

[35] Mohammed KA, Molyneux DH, Albonico M, Rio F (2006). Progress towards eliminating lymphatic filariasis in Zanzibar: a model programme. Trends Parasitol.

[36] Pedersen EM, Stolk WA, Laney SJ, Michael E (2009). The role of monitoring mosquito infection in the Global Programme to Eliminate Lymphatic Filariasis. Trends Parasitol.

[37] Irish SR, Moore SJ, Derua YA, Bruce J, Cameron MM (2013). Evaluation of gravid traps for the collection of *Culex quinquefasciatus*, a vector of lymphatic filariasis in Tanzania. Trans R Soc Trop Med Hyg.

[38] Addiss DG, Brady MA (2007). Morbidity management in the Global Programme to Eliminate Lymphatic Filariasis: a review of the scientific literature. Filar J.

[39] Bernhard P, Magnussen P, Lemnge MM (2001). A randomized, double-blind, placebo-controlled study with diethylcarbamazine for the treatment of hydrocele in an area of Tanzania endemic for lymphatic filariasis. Trans R Soc Trop Med Hyg.

[40] Eddy BA, Blackstock AJ, Williamson JM, Addiss DG, Streit TG, de Rochars VMB, Fox LM (2014). A longitudinal analysis of the effect of mass drug administration on acute inflammatory episodes and disease progression in lymphedema patients in Leogane, Haiti. Am J Trop Med Hyg.

[41] Kisoka WL, Simonsen PE, Malecela MN, Tersboel BP, Mushi DL, Meyrowitsch DW: **Factors influencing drug uptake during mass drug administration for control of lymphatic filariasis in rural and urban Tanzania.** *PLoS One* 2014, **10:**e109316 10.1371/journal.pone.0109316 PMC4190414 25296034

[42] Krentel A, Fischer PU, Weil GJ (2013). A review of factors that influence individual compliance with mass drug administration for elimination of lymphatic filariasis. PLoS Negl Trop Dis.

[43] Jordan P (1956). Filariasis in the Eastern, Tanga and Northern Provinces of Tanganyika. East Afr Med J.

[44] Kyelem D, Sanou S, Boatin B, Medlock J, Coulibaly S, Molyneux DH (2003). Impact of long-term ivermectin (Mectizan®) on *Wuchereria bancrofti* and *Mansonella perstans* infections in Burkina Faso: strategic and policy implications. Ann Trop Med Parasitol.

[45] Keylem D, Medlock J, Sanou S, Bonkoungou M, Boatin B, Molyneux DH (2005). Short communication: Impact of long-term (14 years) bi-annual ivermectin treatment on *Wuchereria bancrofti* microfilaraemia. Trop Med Int Health.

[46] Mwakitalu ME, Malecela MN, Pedersen EM, Mosha FW, Simonsen PE (2013). Urban lymphatic filariasis in the city of Tanga, Tanzania, after seven rounds of mass drug administration. Acta Trop.

[47] Njenga SM, Mwandawiro CS, Wamae CN, Mukoko DA, Omar AA, Shimada M, Bockarie MJ, Molyneux DH (2011). Sustained reduction in prevalence of lymphatic filariasis infection in spite of missed rounds of mass drug administration in an area under mosquito nets for malaria control. Parasit Vectors.

[48] Kelly-Hope LA, Molyneux DH, Bockarie MJ (2013). Can malaria vector control accelerate the interruption of lymphatic filariasis transmission in Africa; capturing a window of opportunity?. Parasit Vectors.

[49] Reimer LJ, Thomsen EK, Tssch DJ, Henry-Halldin CN, Zimmerman PA, Baea ME, Dagoro H, Sasapu M, Hetzel MW, Bockarie MJ, Michael E, Siba PM, Kazura JW (2013). Insecticidal bed nets and filariasis transmission in Papua New Guinea. N Engl J Med.

[50] Richards FO, Emukah E, Graves PM, Nkwocha O, Nwankwo L, Rakers L, Mosher A, Patterson A, Ozaki M, Nwoke BEB, Ukaga CN, Njoku C, Nwodu K, Obasi A, Miri ES (2013). Community-wide distribution of long-lasting insecticidal nets can halt transmission of lymphatic filariasis in Southeastern Nigeria. Am J Trop Med Hyg.

